# The impact of adolescent physical activity on quality of life: a moderated mediation model

**DOI:** 10.3389/fpubh.2025.1625066

**Published:** 2025-07-18

**Authors:** Sen Ma, Yan Gao, Liangyu Zhao, Wenze Sui, Yuke Yang, Yining Hu

**Affiliations:** School of Physical Education, Shandong University, Jinan, China

**Keywords:** physical activity, anxiety, quality of life, gender, adolescents

## Abstract

**Background:**

Quality of Life (QoL) is a crucial multi-dimensional structure in the development process of adolescents. As a key topic in adolescent research, existing achievements all indicate that participating in physical activities can improve the QoL of adolescents. However, the underlying mechanisms—the mediating role of anxiety and the underlying gender differences—remain insufficiently understood.

**Purpose:**

This study investigates the mechanisms by which adolescent physical activity influences QoL. Specifically, it examines the mediating role of anxiety and explores gender as a moderating factor. A multidimensional analysis of QoL outcomes is also conducted to provide a more nuanced understanding.

**Methods:**

We analyzed cross-sectional data from 9,504 adolescents across 17 cities in Shandong Province, China. The collected data were statistically analyzed using SPSS 27.0. Build a moderated mediation model using the PROCESS macro of SPSS.

**Results:**

(1) There is a significant positive correlation between teenagers’ participation in sports and their QoL (*β* = 4.243, *p* < 0.01). (2) Anxiety plays a key mediating role between teenagers’ participation in sports and their QoL (*β* = 0.594, *p* < 0.05). (3) Gender plays a significant positive moderating role between adolescent sports participation and QoL (*β* = 0.781, *p* < 0.01). (4) Gender plays a significant positive moderating role between anxiety and QoL (*β* = 1.214, *p* < 0.05). (5) Gender plays a significant positive moderating role in the relationships between anxiety and living environment (*β* = 0.536, *p* < 0.01).

**Conclusion:**

Teenagers’ participation in sports can directly or indirectly improve their QoL by reducing anxiety. Meanwhile, gender plays a moderating role in it. Furthermore, gender plays a significant moderating role in the relationships between anxiety and psychosocial function, anxiety and physical and mental health, anxiety and living environment, and exercise participation and physical and mental health. These findings emphasize the importance of adopting gender-specific strategies in physical intervention measures to improve the QoL of adolescents.

## 1 Introduction

According to the definition given by the World Health Organization (WHO), Quality of Life (QoL) encompasses an individual’s perception of their living standards and status within the realm of their life culture and values, as well as their life goals, future life, living standards and concerns (1). Among adolescents, QoL serves as a critical indicator of developmental well-being, shaped by both maturational processes and situational life events, and represents a key outcome in public health and healthcare promotion initiatives (2, 3). The number of adolescents worldwide has soared to a record level, reaching 1.3 billion, which constitutes approximately one-sixth of the global population. This number is expected to keep rising through 2050, particularly in low- and middle-income nations, where nearly 90% of individuals aged 10 to 19 reside (4).

Adolescent QoL not only influences immediate outcomes such as academic performance and social adaptability (5), but also exerts long-term effects on future mental health and psychosocial functioning (6). For this population, QoL extends beyond physical health and scholastic achievement to include psychological well-being (7), social adaptation, and overall life satisfaction (5, 8). In the Chinese research context, a well-established multidimensional QoL model for adolescents incorporates four core domains: psychosocial functioning, physical and psychological health, environmental quality, and satisfaction with life (9). Numerous scholars emphasize that identifying and understanding the determinants of adolescent QoL is essential for developing targeted intervention strategies (10). Recent efforts in this field have focused on the early detection of risk and protective factors, the design of evidence-based interventions, and the assessment of both short- and long-term benefits (11, 12). Nevertheless, from a personalized intervention standpoint, there remains a pressing need to develop and evaluate more effective strategies that can be tailored to adolescents across different ages, sexes, and sociocultural backgrounds. Therefore, this study aims to investigate the key mediating role of anxiety in the relationship between physical activity and adolescent well-being, while also evaluating the potential moderating effect of gender on this mediation pathway. Moreover, a multidimensional assessment of QoL has enhanced the overall understanding of the topic.

### 1.1 The relationship between physical activity and quality of life

Among the most compelling and widely studied protective factors is physical activity —an area of growing scholarly attention in recent years (13). Adolescence represents a pivotal transitional stage in personal development (4), during which both consistent physical activity and psychological stability play essential roles (14). More and more evidence shows that improving the level of physical participation has significant benefits for the physical and mental health of teenagers (15). Conversely, low levels of participation have been linked to increased risk for psychological disorders and diminished QoL (16–18). Engaging in regular physical activity not only enhances physical fitness and somatic health (15), but also improves subjective well-being and life satisfaction by fostering a more positive bodily self-perception (19). As such, physical activity represents a crucial avenue for promoting holistic adolescent development and elevating QoL across multiple dimensions.

While preliminary evidence suggests that physical activity may positively influence both physical and psychological well-being, as well as overall quality of life among adolescents (20), current research remains insufficient in elucidating the underlying mechanisms driving these effects. A particularly important but under-researched mechanism is the mediating role of anxiety within this relationship. Specifically, there is a lack of empirical studies that rigorously examine and confirm this mediating pathway, which limits the ability to quantitatively determine the degree to which anxiety accounts for the beneficial impact of physical activity on adolescent quality of life. Examining this mechanism is especially pertinent during adolescence—a critical developmental period for establishing emotional and social competencies that are foundational to mental health. Understanding how physical activity improves quality of life through anxiety mitigation carries significant theoretical and practical implications for designing targeted, evidence-based interventions to promote youth health.

### 1.2 The mediating role of anxiety

Adolescents today face multiple challenges, including academic pressure, social difficulties, and the complexities of physical and mental development, leading to a significant decline in time spent engaging in physical activity (21), and increasingly prominent mental health issues (22). It is estimated that around 14% of the global population between the ages of 10 and 19 is impacted by mental health disorders (23), many of which go unrecognized and receive no treatment. Young people facing such psychological challenges are at a higher risk of encountering social marginalization, prejudice, and stigmatization—which may discourage them from accessing support—as well as struggles in academic settings, engagement in dangerous activities, physical health complications, and breaches of their fundamental human rights (24). Anxiety, as one of the most common psychological problems among adolescents, It is estimated that 4.4% of individuals between the ages of 10 and 14, and 5.5% of those aged 15 to 19, are impacted by anxiety disorders (23). Anxiety not only directly affects their mental health, but may also indirectly influence their QoL (QoL) through a series of psychological and behavioral responses (25, 26). The elevated levels of life stress associated with anxiety disorders may impair psychological health and foster a progressively isolated and sedentary lifestyle (27). Adolescents with high levels of anxiety often exhibit lower life satisfaction and social competence, as well as more pronounced psychological and behavioral issues (28).

Physical exercise is not only a fundamental component of physical health, but also plays a vital role in promoting mental and emotional well-being, ultimately contributing to an enhanced overall QoL (29). Regular participation in physical activities stimulates the brain’s natural release of endorphins—neurochemicals that foster social bonding, reduce pain perception, and improve emotional state (30, 31). Consequently, individuals are likely to experience diminished feelings of anxiety and sadness. Meanwhile, current meta-analytic evidence derived from adolescent and young adult cohorts suggests that consistent engagement in physical activity and a variety of exercise modalities may play a role in mitigating symptoms of depression and anxiety (29, 31). Research from Turkey demonstrates that consistent engagement in sports activities is significantly associated with reduced levels of social anxiety. Higher levels of physical exercise contribute to the reduction of anxiety symptoms and help alleviate the detrimental effects associated with psychological disorders (32).

According to Social Cognitive Theory (SCT), within the domain of sports, sustained participation can significantly enhance adolescents’ self-efficacy during critical developmental periods, particularly in domains such as bodily control, overcoming athletic challenges, and regulating emotional responses—ultimately contributing to a substantial reduction in anxiety (33). Therefore, physical activity serves as a key cognitive-emotional mechanism for alleviating anxiety through the enhancement of both sport-related and emotion-regulation self-efficacy. Moreover, Self-Determination Theory (SDT) posits that engaging in appropriate forms of physical activity effectively fulfills adolescents’ fundamental psychological needs (34). When these essential needs are satisfied, physical activity fosters the development and maintenance of intrinsic motivation, thereby promoting greater willingness and consistency in participation, which establishes a reinforcing cycle that amplifies its enduring efficacy in anxiety reduction (35). Collectively, these theoretical perspectives provide a robust foundation for the central hypothesis of this study: that anxiety mediates the relationship between physical activity engagement and QoL. On the other hand, according to the Hypothalamic–Pituitary–Adrenal (HPA) axis theory, physical activity can reduce the secretion of cortisol by regulating the activity of the HPA axis, thereby alleviating anxiety (36). There is clear neuroendocrine evidence supporting the inhibitory effect of physical activity on anxiety (e.g., normalization of HPA axis function), which provides biological plausibility for the mediating pathway “physical activity → anxiety reduction → QoL improvement” (37, 38).

### 1.3 The moderating role of gender

In addition, it is worth noting that adolescents of different genders, ages, and family backgrounds may exhibit significant differences in physical activity, anxiety levels, and QoL (39, 40). Given that gender-based disparities emerge across multiple domains—including patterns of physical activity, the prevalence of anxiety disorders, and subjective perceptions of QoL (41)—this study integrates gender as a pivotal moderating variable within the mediation framework. Drawing upon Gender Role Theory, the formation of distinct gender roles during adolescence heightens societal expectations regarding gender-specific behaviors and emotional expression (42). For instance, girls are often socially permitted to exhibit anxiety but face greater external judgment, whereas boys are typically expected to mask their emotions while managing heightened performance pressures (43). These divergent socialization processes may shape adolescents’ motivation to engage in physical activity, their sensitivity to and expression of anxiety, and the dimensions of QoL they value most (42, 44). Accordingly, we posit that gender not only influences adolescents’ participation in physical activity, their anxiety levels, and their perceived QoL, but also modulates the degree to which physical activity functions as a pathway for alleviating anxiety and enhancing overall well-being.

Additionally, the study explored a range of demographic factors—including age, family socioeconomic status, and residential context—that may exert influence on the primary variables (45). Crucially, drawing upon gender role theory and developmental research on adolescence, these control variables may exhibit differential effects across genders with respect to participation in physical activity, levels of anxiety, and subjective perceptions of QoL. Consequently, demographic control variables were incorporated into the analytical framework to account for their potential moderating influence when examining the role of gender.

In view of the previously described context, this study centers on adolescents as the focal population and establishes a moderated mediation model. The following research hypotheses are proposed:

*H1*: There is a positive relationship between adolescent physical activity and QoL.

*H2*: Anxiety functions as a mediator in the relationship between adolescent physical activity and QoL.

*H3*: Gender moderates the association between adolescent physical activity and QoL.

*H4*: Gender serves as a moderator in the relationship between adolescent physical activity and anxiety.

*H5*: Gender acts as a moderating factor in the relationship between anxiety and QoL.

**Figure 1:**
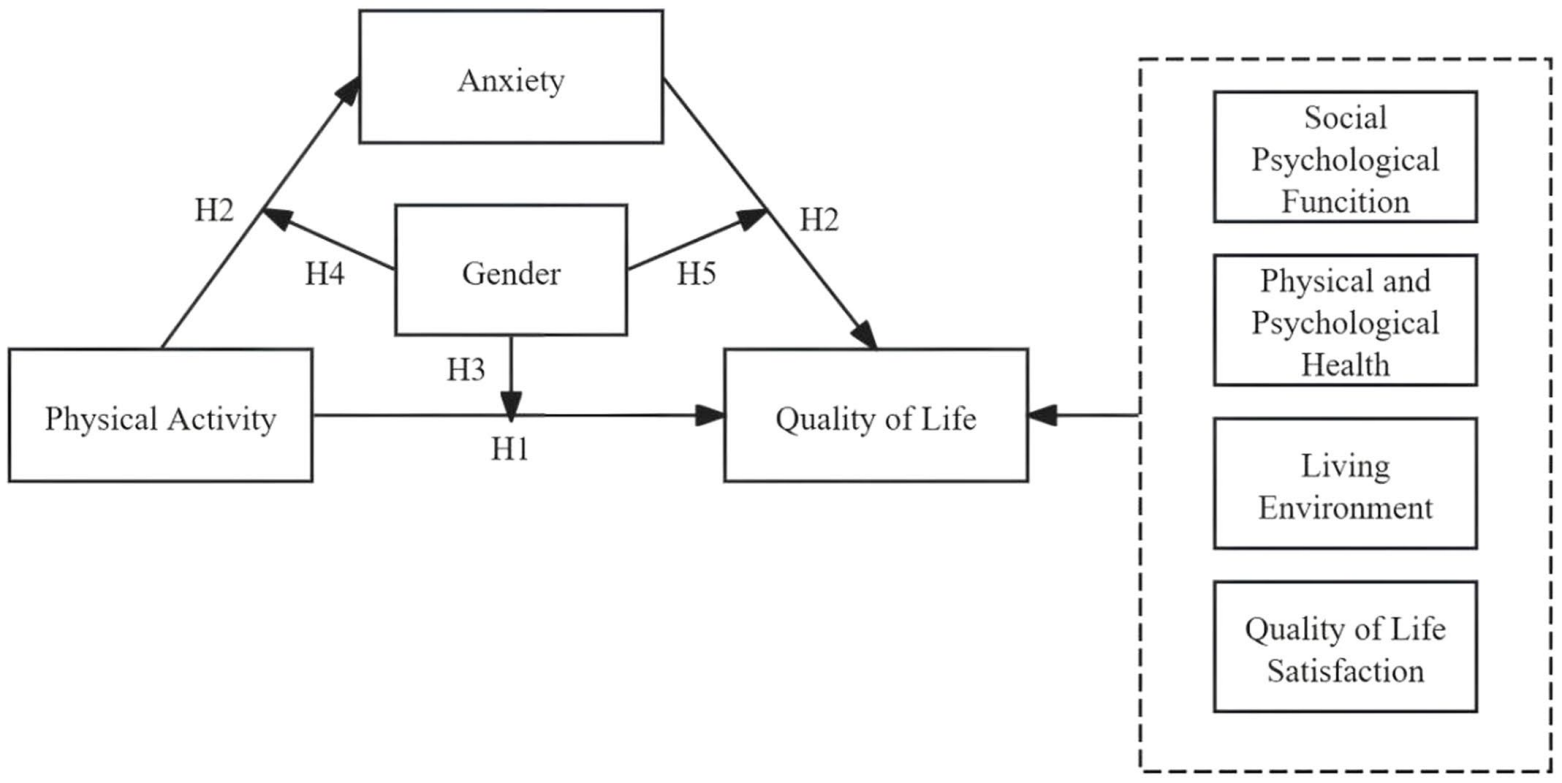
Theoretical hypothesis model.

The hypothesized model is presented in Figure 1.

## 2 Methodology

### 2.1 Participants and procedures

This study was conducted in accordance with the guidelines of the Declaration of Helsinki. All procedures involving human participants were approved by the Ethics Committee of Shandong University (20180517). This study adopted the probability Proportional sampling (PPS) method. Participants were randomly selected from 186 middle and high schools in 16 prefecture-level cities (Jinan, Qingdao, Zibo, Zaozhuang, Dongying, Yantai, Weifang, Jining, Taian, Weihai, Rizhao, Linyi, Dezhou, Liaocheng, Binzhou, Heze) of Shandong Province during 2020–2021. The target sample consisted of adolescents aged 12 to 18 years. The demographic characteristics of the participants are detailed in Table 1. Both the research participants and their parents signed the written informed consent form. The data collection process strictly adheres to standardized protocols: researchers who have undergone two rounds of standardized training provide on-site guidance for students to complete the questionnaire filling. The entire research process adheres to the principles of anonymization and confidentiality.

**Table 1:** Sociodemographic characteristics of the participants and descriptive statistics for main variables.

Variables	Definition	Frequency	Percentage (%)	Mean	SD	Skewness	Kurtosis
Gender	Male	4,533	47.70	1.523	0.499	−0.092	−1.992
	Female	4,971	52.30			−0.124	−1.985
Address	Urban	4,457	46.90	1.531	0.499	−0.908	−1.175
	Non-urban	5,047	53.10			0.945	−0.711
Only child	Yes	2,787	29.32	1.707	0.455	1.274	0.034
	No	6,717	70.68			−0.378	3.691
Father’s education level	Below high school	5,903	62.11	1.560	0.780	0.045	0.193
	From high school to undergraduate	1882	19.80			1.896	4.348
	Bachelor’s degree or above	1719	18.09			1.366	2.494
Mother’s education level	Below high school	6,605	69.50	1.451	0.735	−0.092	−1.992
	From high school to undergraduate	1,510	15.89			−0.124	−1.985
	Bachelor’s degree or above	1,389	14.61			−0.908	−1.175
Family economic condition	Very difficult	310	3.26	2.900	0.608	0.945	−0.711
	Rather difficult	1,190	12.52			1.274	0.034
	Medium	7,299	76.80			−0.378	3.691
	Relatively wealthy	548	5.77			0.045	0.193
	Very rich	157	1.65			1.896	4.348
Quality of life	Continuous variable	9,504		136.070	20.033	1.366	2.494
Anxiety	Continuous variable	9,504		1.558	0.601	−0.092	−1.992
Physical activity	Continuous variable	9,504		3.975	1.352	−0.124	−1.985

### 2.2 Measures

#### 2.2.1 Physical activity questionnaire for adolescents (PAQ-A)

This study assessed the physical activity levels of adolescents using the revised Physical Activity Questionnaire for Adolescents (PAQ-A), originally developed by Li Xin et al. (46). This scale consists of nine items designed to evaluate adolescents’ daily physical activity levels over the past week. It also investigates whether any unusual circumstances arose during this period that could have affected their usual participation in physical activities. All questions are evaluated based on a five-point Likert scale. The overall score is determined by averaging the values of all items, where higher scores signify increased physical activity levels. Specifically, a score exceeding 3 indicates high physical activity levels, scores between 2 and 3 suggest moderate participation, and scores below 2 signify low involvement. To test the reliability and validity of SCL-90, exploratory factor analysis was used to test the validity of the scale (KMO = 0.868, *p* = 0.000), and Cronbach’s alpha was used to test its reliability (Cronbach’s alpha = 0.930) (47).

#### 2.2.2 The QoL scale for children and adolescents (QLSCA)

The QoL Scale for Children and Adolescents (QLSCA), developed by Wu Hanrong and colleagues, was employed to assess the QoL among adolescents (10). The QLSCA comprises 49 items, encompassing four main domains: physical and mental health, psychosocial functioning, living environment, and satisfaction with QoL. It covers 13 particular aspects, such as the interaction between teachers and students, relationships among peers, the dynamics within parent–child interactions, competence in learning along with associated attitudes, one’s self-perception, overall physical health, experiences of negative emotions, perspectives on homework, the ease of daily living, chances for participating in activities, athletic proficiency, and a sense of personal satisfaction. Each item on the scale is rated on a four-point Likert scale (1 = never; 2 = rarely; 3 = often; 4 = always), where lower scores indicate a poorer QoL and higher scores indicate a better QoL. The scale contains 13 negatively worded items. The total score is calculated by summing the scores for all items. To test the reliability and validity of SCL-90, exploratory factor analysis was used to test the validity of the scale (KMO = 0.965, *p* = 0.000), and Cronbach’s alpha was used to test its reliability (Cronbach’s alpha = 0.939) (48).

#### 2.2.3 The symptom checklist-90 (SCL-90)

Anxiety levels were assessed through the Symptom Checklist-90 (SCL-90), a psychological measurement instrument created by Derogatis and his team (49). The SCL-90 consists of 90 items across 9 factors, with item content derived primarily from psychopathological symptomatology. It encompasses a wide range of domains, including sensory experiences, cognition, affect, behavior, interpersonal relationships, daily habits, diet, and sleep. It is commonly used to evaluate mental health status and behavioral problems. This study specifically employed the Anxiety subscale, one of the nine factors, comprising 10 items (items 2, 17, 23, 33, 39, 57, 72, 78, 80, and 86). These items indicate psychological symptoms and experiences with a clinical association to anxiety disorders. Each item is scored on a four-point scale. The final score is calculated as the average across all items. A score above 2 implies the existence of anxiety symptoms, whereas a score of 2 or less signifies their nonexistence. To test the reliability and validity of SCL-90, exploratory factor analysis was used to test the validity of the scale (KMO = 0.948, *p* = 0.000), and Cronbach’s alpha was used to test its reliability (Cronbach’s alpha = 0.911) (50).

#### 2.2.4 Socio-demographic variables

This study included several socio-demographic factors as control variables, including gender, family structure, parental education level, household economic status, and residential location. Gender served as a moderating variable and was coded as a dummy variable (1 = male, 2 = female). Family structure was defined by whether the adolescent was an only child. Only-child status was coded as 1; non-only-child status was coded as 2. Parental education level was measured based on the highest level of education attained (excluding adult education), coded as follows: below high school = 1, high school to undergraduate level = 2, above undergraduate = 3. Household economic status was categorized into five levels: 1 = very poor, 2 = relatively poor, 3 = average, 4 = relatively affluent, 5 = very affluent. Based on the actual construction situation, the location of the residence is classified as urban (code 1) or non-urban (code 2).

### 2.3 Statistical analyses

Initially, the dataset was examined to confirm its authenticity, validity, and alignment with the study’s requirements. A power analysis performed with G*Power 3.1 revealed that at least 103 participants were required to detect the intended effect size with 80% statistical power and a significance threshold of.05. The obtained sample size of 9,504 clearly exceeded this minimum criterion (51). Subsequently, the required variables were extracted and documented using the specified tools. All analyses were conducted using SPSS 27.0 software (IBM SPSS Inc., Chicago, USA). The statistical methods employed included evaluations for common method bias, descriptive statistics, correlation analysis, and analysis of variance (ANOVA). To remove the impact caused by the dimensionality differences in the independent variable, all variables were standardized before performing the mediation analysis. To investigate the mediating influence of anxiety in the relationship between adolescents’ physical activity and QoL, Model 4 of the SPSS PROCESS macro was utilized. A bias-corrected bootstrap percentile approach was applied to calculate 95% confidence intervals (CIs). If the CI for a path coefficient did not encompass zero, the mediating effect was deemed statistically significant. Lastly, Model 59 of the PROCESS macro was applied to evaluate the moderating role of gender within the mediation framework.

## 3 Results

### 3.1 Common method bias test

The Harman single-factor test was employed to assess the presence of common method bias (52). The analysis extracted 12 factors with eigenvalues exceeding 1, where the largest factor explained 21.16% of the total variance, which is substantially lower than the 40% criterion. Consequently, these findings suggest that common method bias does not pose a substantial issue in this study.

### 3.2 Descriptive statistics

Table 1 displays the descriptive statistical measures, inter-variable correlations, and reliability estimates for the variables under investigation. Analysis revealed that the distribution of all variables deviated from normality, as evidenced by kurtosis and skewness values that surpassed the conventional thresholds (53). The sample comprised a total of 9,504 participants, among whom females accounted for a slightly larger proportion (52.30%) than males (47.70%). Over half of the participants originated from non-urban regions (53.10%), and the majority were not only children (70.68%). Furthermore, most participants reported that their families had a moderate economic situation (76.80%), and their parents generally possessed relatively low levels of educational attainment, with 62.11% for fathers and 69.50% for mothers.

### 3.3 Analysis of variance

The results of the analysis of variance of the main variables are shown in Supplementary Table 1. Female students scored significantly lower than male students in terms of physical activity and QoL (*p* < 0.01), but exhibited significantly higher scores in anxiety (*p* < 0.01). Furthermore, teenagers residing in urban areas exhibited markedly greater levels of physical activity and QoL compared to their counterparts in non-urban settings (*p* < 0.01) and significantly lower anxiety scores (*p* < 0.01). Regarding only-child status, no notable difference was found in QoL scores between children without siblings and those with siblings (*p* > 0.05). However, children without siblings demonstrated markedly higher physical activity levels and reduced anxiety levels in comparison to children with siblings (*p* < 0.01). In terms of parental education, adolescents whose parents have completed high school, undergraduate, or higher levels of education showed markedly higher scores in QoL and physical activity, as compared to those whose parents did not finish high school (*p* < 0.01). In contrast, they had significantly higher anxiety scores. Finally, with respect to family economic condition, scores for QoL and physical activity increased with improvements in economic conditions, while anxiety scores decreased, all showing statistically significant differences (*p* < 0.01).

It should also be emphasized that, with the exception of family economic condition, the remaining control variables demonstrate limited clinical significance in explaining the observed variations in the outcomes (*η*^2^ < 0.015). Conversely, family economic condition exhibited a moderate effect in explaining variations in quality of life (*η*^2^ = 0.049), representing roughly 4.9% of the total variance. It indicates that there is a meaningful relationship between the family economic condition and an individual’s QoL (See Supplementary Table 2).

**Figure 2:**
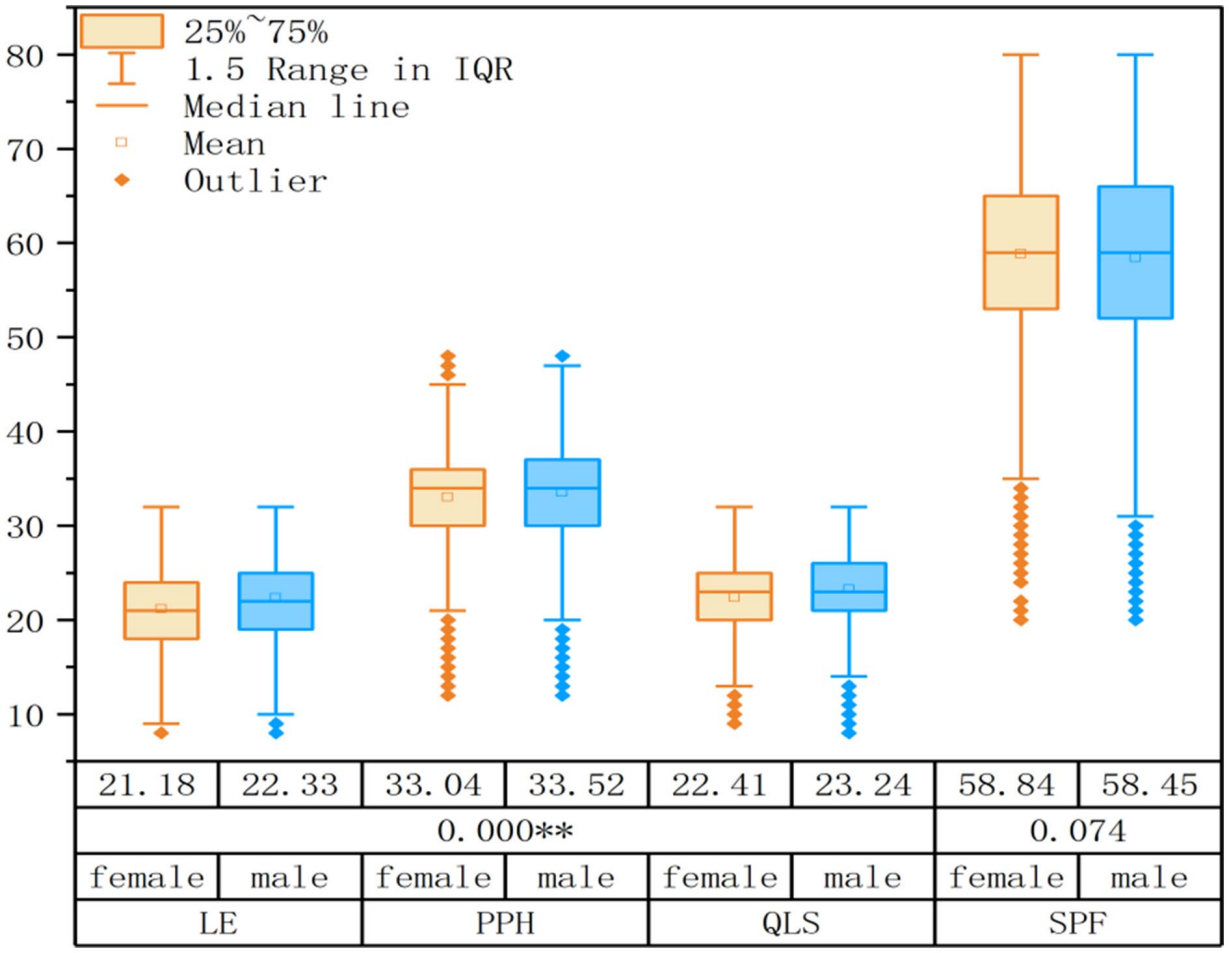
Multi-dimensional box plot of quality of life. SPF, Social Psychological Function; PPH, Physical and Psychological Health; LE, Living Environment; QLS, Quality of Life Satisfaction.

An analysis of variance was conducted for the four dimensions of QoL. As shown in Figure 2, significant differences based on gender were evident in the subsequent dimensions: Physical and Psychological Health (*F* = 13.445, *p* < 0.01), Living Environment (*F* = 144.611, *p* < 0.01), and QoL Satisfaction (*F* = 82.158, *p* < 0.01), with males scoring higher than females. However, no significant gender difference was observed in Social Psychological Function (*F* = 3.19, *p* = 0.074).

### 3.4 Correlation analysis

The correlation analysis results for the key variables are summarized in Table 2. The mean values of the key variables are summarized below: QoL (136.070 ± 20.033), adolescent physical activity (3.975 ± 1.352), and anxiety (1.558 ± 0.601). Pairwise correlations indicated statistically significant relationships: QoL showed a positive association with physical activity (*R* = 0.298, *p* < 0.01); Anxiety exhibited a negative relationship with QoL (*R* = −0.514, *p* < 0.01); Physical activity had a weak negative correlation with anxiety (*R* = −0.095, *p* < 0.01). These findings align well with theoretical expectations.

**Table 2:** Correlation analysis of major variables.

Variables	Correlation								
	1	2	3	4	5	6	7	8	9
1. Gender	1								
2. Address	0.044**	1							
3. Only child	0.176**	0.204**	1						
4. Father’s education level	−0.047**	−0.309**	−0.208**	1					
5. Mother’s education level	−0.031**	−0.308**	−0.259**	0.616**	1				
6. Family economic condition	0.004	−0.115**	−0.058**	0.172**	0.189**	1			
7. Quality of life	−0.049**	−0.102**	−0.012	0.119**	0.112**	0.165**	1		
8. Anxiety	0.074**	0.040**	0.030**	−0.073**	−0.071**	−0.098**	−0.506**	1	
9. Physical activity	−0.206**	−0.033**	−0.073**	0.050**	0.057**	0.076**	0.299**	−0.095**	1

*p < 0.05, **p < 0.01.

### 3.5 Hierarchical regression and mediation test

To test Hypotheses 1 (H1) and 2 (H2), Model 4 of the SPSS PROCESS macro was applied. To avoid multicollinearity between interaction terms, all independent and moderating variables were standardized. Five socio-demographic variables were included as control variables. QoL was divided into four dimensions for use in hierarchical regression and mediation analysis. The regression analysis outcomes are displayed in Table 3, while the mediation test results are summarized in Table 4.

**Table 3:** Results of hierarchical regression analysis.

Variables	Quality of life			Anxiety			Quality of life		
	*B*	SE	*t*	*B*	SE	*t*	*B*	SE	*t*
Constant	104.109**	1.586	65.655	1.986**	0.050	39.525	135.186**	1.486	90.970
Only child	2.104**	0.443	4.747	0.005	0.014	0.341	2.179**	0.385	5.660
Father’s education level	1.377**	0.319	4.323	−0.027**	0.010	−2.710	0.949**	0.277	3.429
Mother’s education level	0.901**	0.342	2.632	−0.021	0.011	−1.944	0.571	0.297	1.920
Family economic condition	4.082**	0.325	12.573	−0.078**	0.010	−7.606	2.857**	0.283	10.104
address	−2.474**	0.415	−5.964	0.010	0.013	0.765	−2.316**	0.360	−6.430
Physical activity	4.243**	0.143	29.617	−0.038**	0.005	−8.360	3.649**	0.125	29.223
Anxiety							−15.650**	0.281	−55.642
*R* ^2^	0.122			0.021			0.338		
*F*	*F* (6,9497) = 219.783, *p* = 0.000			*F* (6,9497) = 33.483, *p* = 0.000			*F* (7,9496) = 692.064, *p* = 0.000		

*N* = 9,504, *p < 0.05, **p < 0.01.

**Table 4:** Test of the mediating effect of anxiety.

Options	Effect	Effect size	95% CI	Effect ratio
PA → Anxiety→QoL	Total effect	4.243**	[3.962, 4.524]	13.996%
	Direct effect	3.649**	[3.404, 3.894]	
	Indirect effect	0.594**	[0.025, 0.050]	
PA → Anxiety→SPE	Total effect	1.607**	[1.456, 1.758]	14.429%
	Direct effect	1.375**	[1.233, 1.517]	
	Indirect effect	0.232**	[0.020, 0.038]	
PA → Anxiety→PPH	Total effect	0.279**	[0.184, 0.374]	59.128%
	Direct effect	0.114*	[0.027, 0.201]	
	Indirect effect	0.165**	[0.021, 0.045]	
PA → Anxiety→LE	Total effect	1.342**	[1.280, 1.404]	6.102%
	Direct effect	1.260**	[1.201, 1.319]	
	Indirect effect	0.082**	[0.015, 0.029]	
PA → Anxiety→QLS	Total effect	0.941**	[0.878, 1.005]	13.271%
	Direct effect	0.816**	[0.760, 0.873]	
	Indirect effect	0.125**	[0.024, 0.047]	

QoL, Quality of Life; SPF, Social Psychological Function; PPH, Physical and Psychological Health; LE, Living Environment; QLS, Quality of Life Satisfaction; PA, physical activity. *p < 0.05, **p < 0.01.

As illustrated in Table 3, physical activity was identified as a significant positive factor for QoL (*β* = 4.243, *p* < 0.01), which aligns with H1. Moreover, physical activity significantly and negatively influenced anxiety levels (*β* = −0.038, *p* < 0.01), and anxiety also significantly and negatively affected QoL (*β* = −15.650, *p* < 0.01). These findings suggest that adolescents experience both direct improvements in QoL through physical activity and indirect benefits due to reduced anxiety. To evaluate the extent of the mediating effect, the bias-corrected percentile bootstrap method was applied (54).

As shown in Table 4, the mediating role of anxiety demonstrated an effect size of 0.594, with a 95% confidence interval spanning from (0.025, 0.050), which does not include zero—indicating a significant mediation effect. The indirect influence of physical activity on QoL mediated by anxiety constituted 13.996% of the total effect, thereby supporting H2.

### 3.6 Moderating effect of gender

To test the moderating role of gender, Model 59 of the SPSS PROCESS macro was applied. Five socio-demographic variables were controlled for, and all predictor variables were standardized to examine the moderated mediation model. As indicated in Table 5, gender had a significant moderating effect on the associations between physical activity and QoL (*β* = 0.781, *p* < 0.01) and between anxiety and QoL (*β* = 1.214, *p* < 0.05), lending support to Hypotheses 3 (H3) and 5 (H5). In contrast, gender did not significantly influence the relationship between physical activity and anxiety (*β* = 0.006, *p* > 0.05), thus failing to support Hypothesis 4 (H4). The results for the moderating effect of gender are shown in Table 5.

**Table 5:** Test on the moderating effect of gender.

Variables	Quality of life				Anxiety			
	*β*	SE	*t*	*p*	*β*	SE	*t*	*p*
Constant	140.634	2.577	54.572	0.000**	1.925	0.074	25.860	0.000**
Gender	−3.582	1.437	−2.493	0.013*	0.043	0.039	1.102	0.270
Anxiety	−17.592	0.912	−19.292	0.000**				
Physical activity	2.645	0.384	6.885	0.000**	−0.042	0.014	−3.010	0.003**
Only child	1.957	0.390	5.022	0.000**	−0.007	0.014	−0.518	0.605
Father’s education level	0.978	0.276	3.536	0.000**	−0.026	0.010	−2.599	0.009**
Mother’s education level	0.545	0.297	1.834	0.067	−0.023	0.011	−2.101	0.036*
Family economic condition	2.804	0.283	9.920	0.000**	−0.080	0.010	−7.761	0.000**
Address	−2.301	0.360	−6.394	0.000**	0.010	0.013	0.726	0.468
Physical activity * Gender	0.781	0.259	3.021	0.003**	0.006	0.009	0.687	0.492
Anxiety*Gender	1.214	0.562	2.161	0.031*				
*R* ^2^	0.340				0.024			
*F*	*F* (10,9493) = 488.623, *p* = 0.000				*F* (8,9495) = 28.918, *p* = 0.000			

*N* = 9,504, *p < 0.05, **p < 0.01.

**Figure 3:**
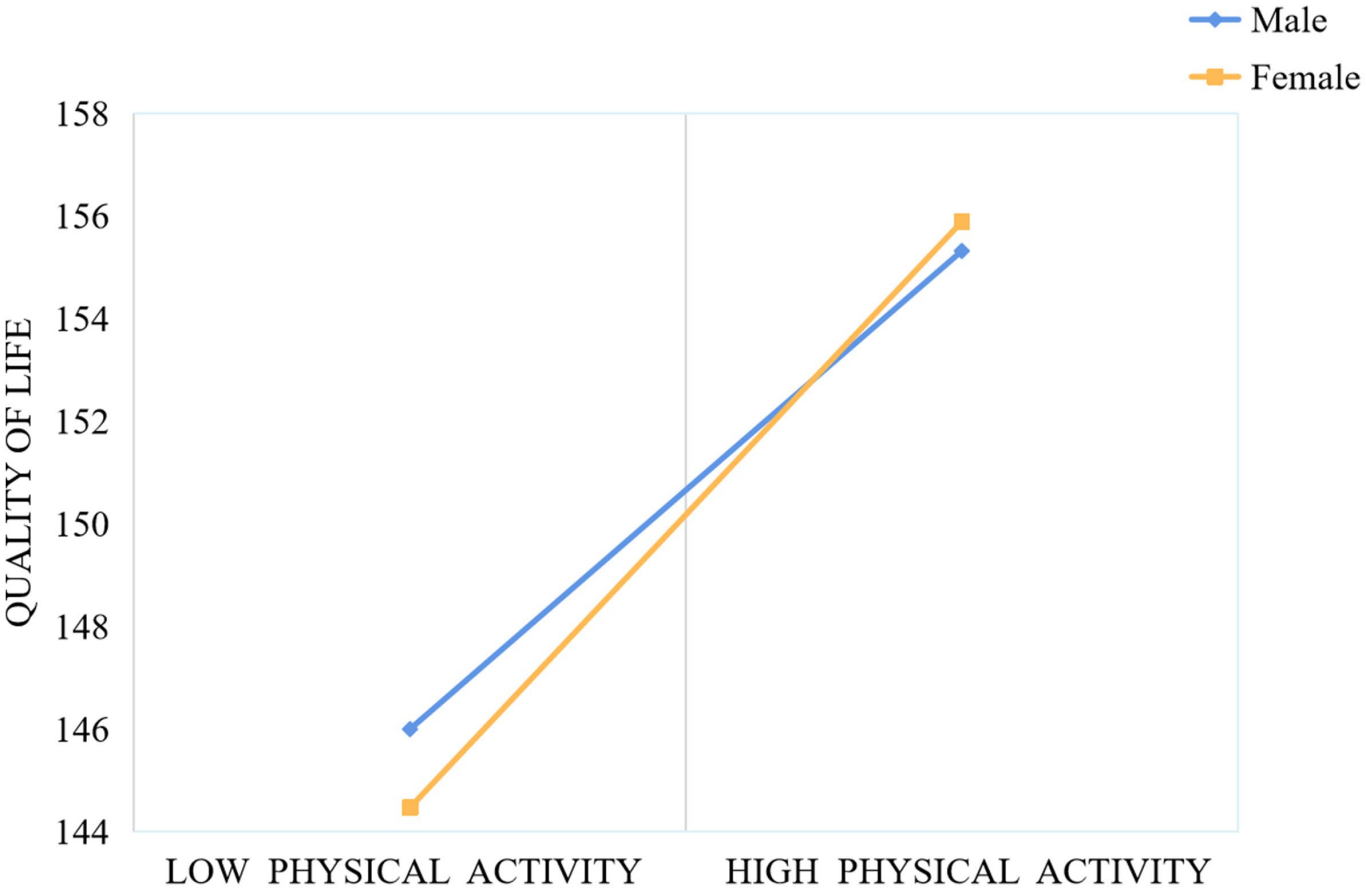
The moderating effect of gender on physical activity and quality of life.

**Figure 4:**
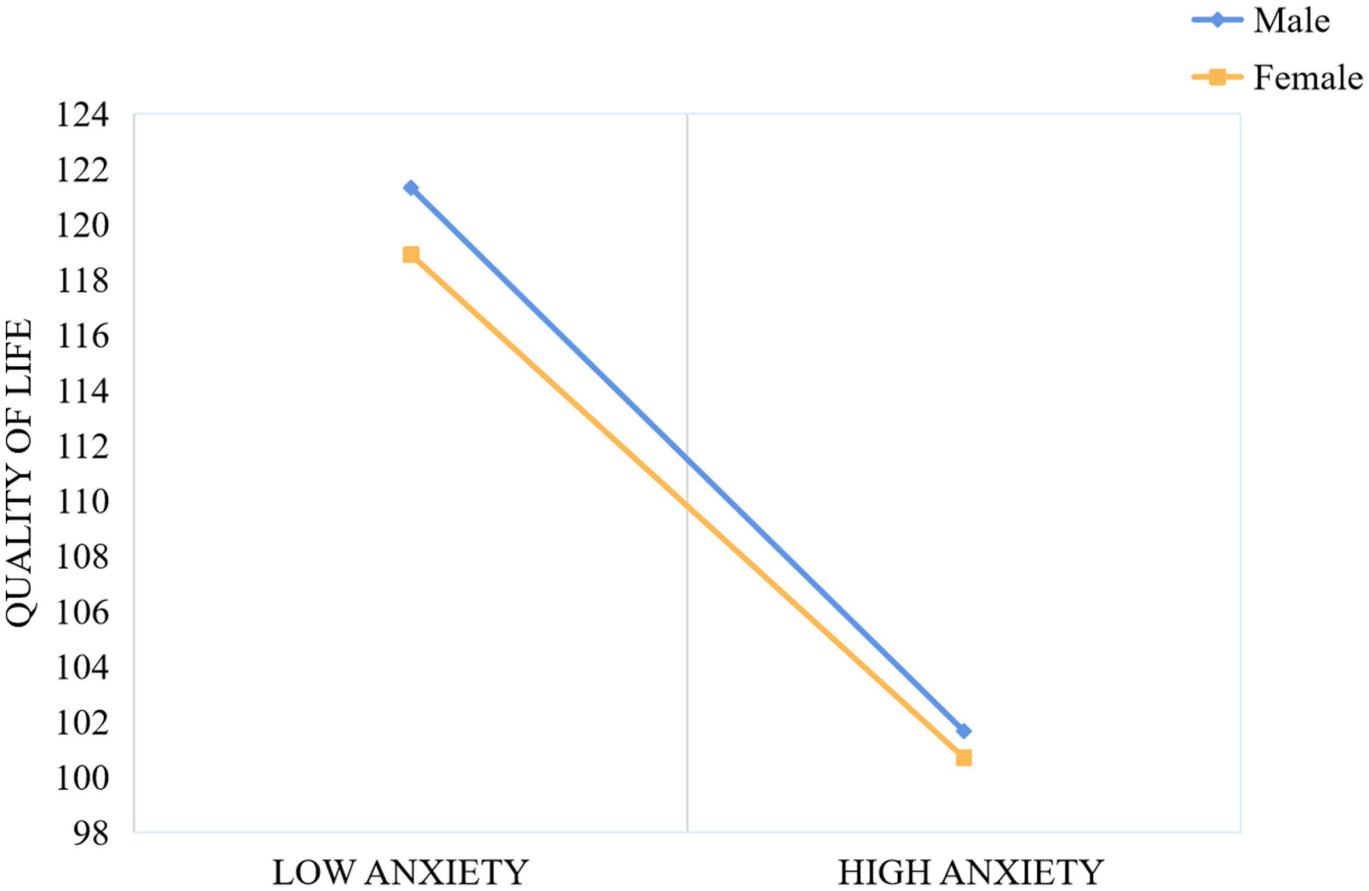
The moderating effect of gender on anxiety and quality of life.

In order to better examine the moderating effect of gender within the mediation model, the sample was divided into male and female groups using standardized gender scores. The relationships between physical activity and QoL, as well as between anxiety and QoL, were assessed individually for each group. As shown in Figure 3, for both male and female adolescents, higher levels of physical activity were associated with greater improvements in QoL, with the effect being more pronounced for females. Similarly, as illustrated in Figure 4, higher anxiety levels were associated with a diminished QoL in both males and females; nonetheless, the detrimental effect of anxiety on QoL was considerably more pronounced in males.

### 3.7 Results of the moderated mediation model

**Figure 5:**
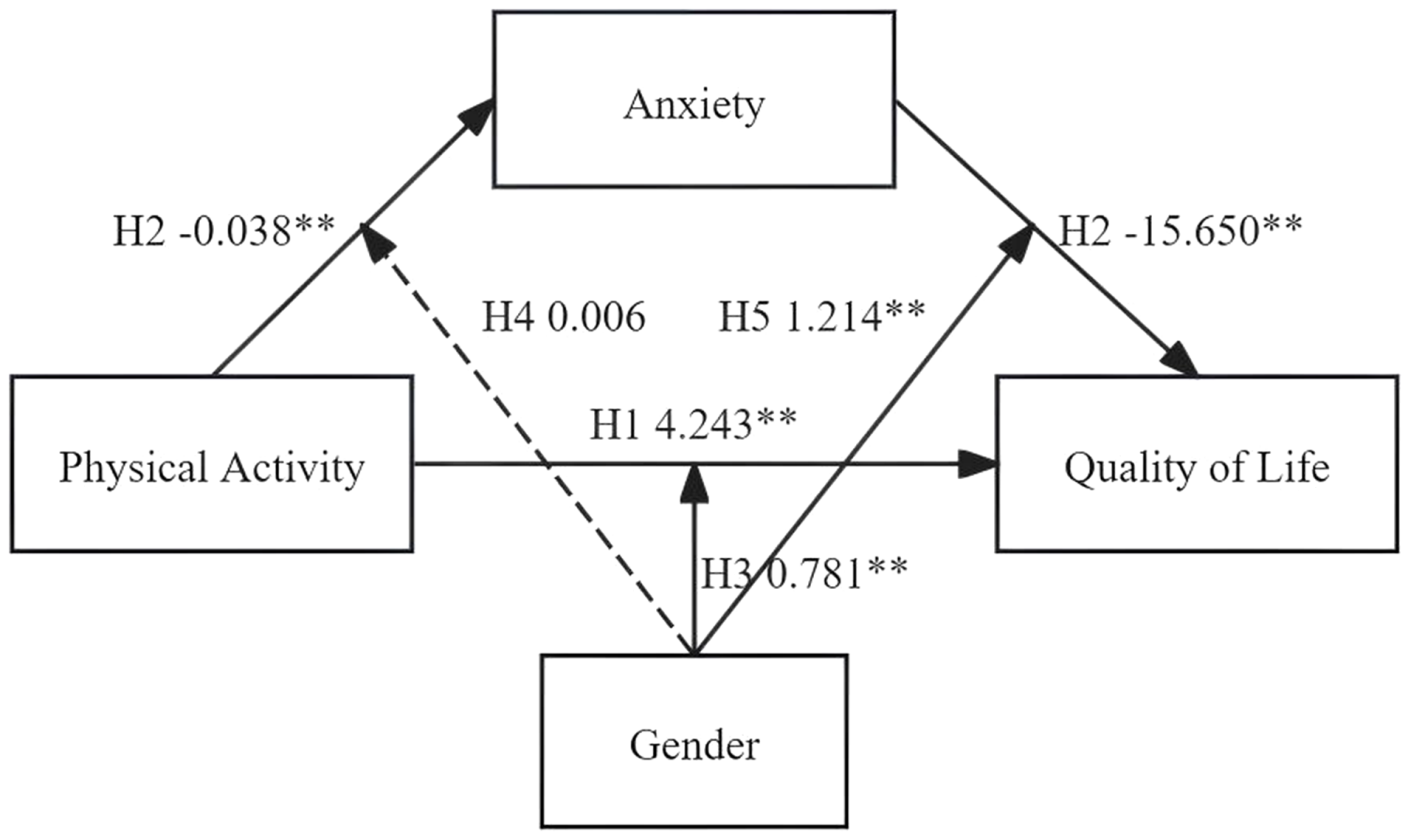
The results of adjusting the mediation model. *p < 0.05; **p < 0.01.

As shown in Table 3, there was a significant positive association between physical activity and QoL (*β* = 4.243, *p* < 0.01), which aligns with Hypothesis 1 (H1). Moreover, anxiety played a significant mediating role in the relationship between physical activity and QoL (*β* = 0.594, *p* < 0.05), providing additional evidence for Hypothesis 2 (H2). A significant positive moderating effect of gender was found between physical activity and QoL (*β* = 0.781, *p* < 0.01), supporting Hypothesis 3 (H3). Gender also significantly moderated the relationship between anxiety and QoL (*β* = 1.214, *p* < 0.05), supporting Hypothesis 5 (H5). However, gender did not exert a statistically significant moderating effect on the relationship between physical activity and anxiety (*β* = 0.006, *p* > 0.05), thus Hypothesis 4 (H4) was not supported. The overall moderated mediation model is illustrated in Figure 5.

### 3.8 Multidimensional analysis of quality of life

**Figure 6:**
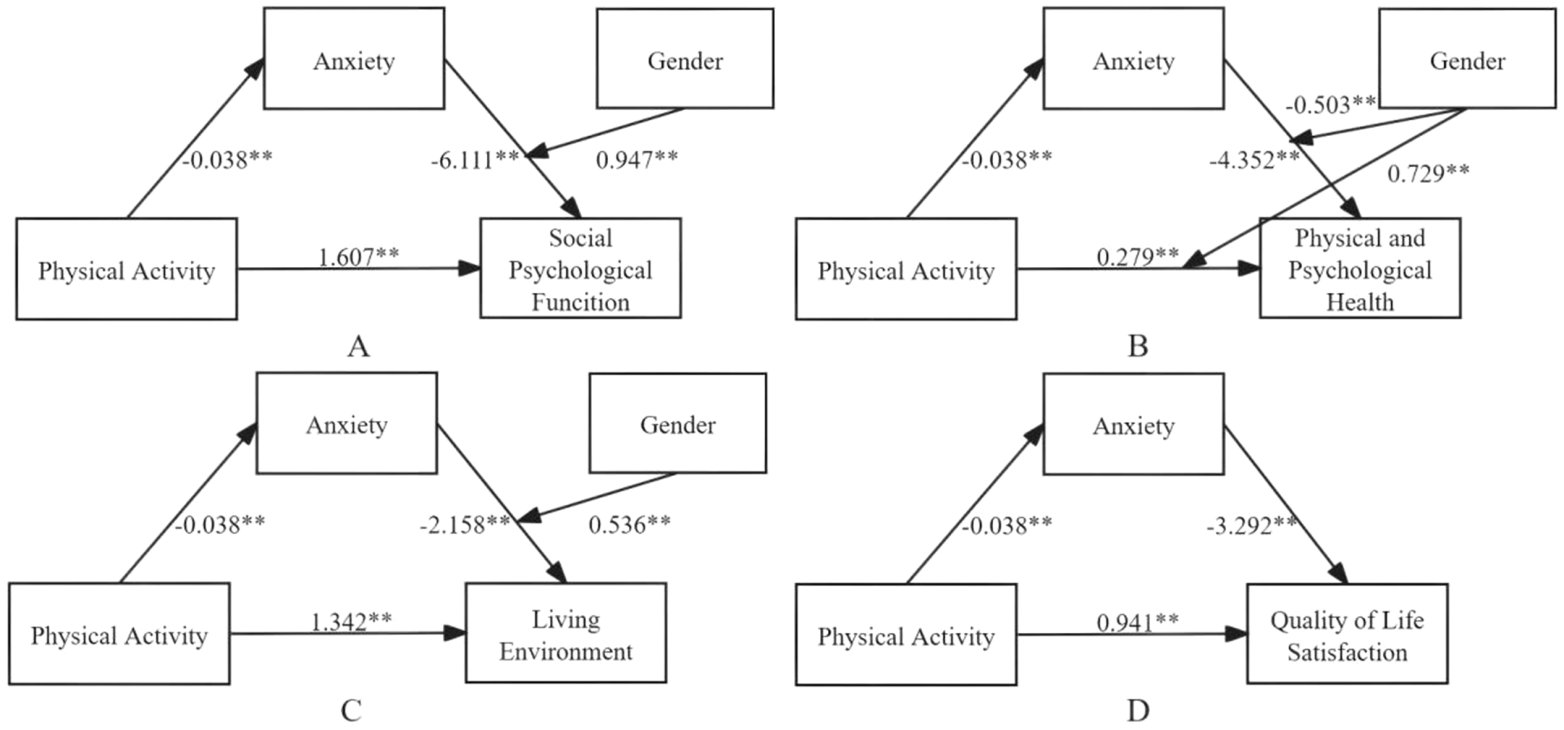
Results of the multi-dimensional moderating mediation model for quality of life. *p < 0.05; **p < 0.01.

In the present study, QoL was divided into four separate dimensions. Hierarchical regression analysis and mediation testing were performed using Model 4 of the SPSS PROCESS macro. The findings of the hierarchical regression analysis are provided in the Supplementary Tables 2–6, while mediation testing results are shown in Table 4. As presented in the Supplementary Tables 2–6, physical activity significantly and positively predicted four dimensions of QoL: Social Psychological Function (*β* = 1.607, *p* < 0.01), Physical and Psychological Health (*β* = 0.279, *p* < 0.01), Living Environment (*β* = 1.342, *p* < 0.01), and QoL Satisfaction (*β* = 0.941, *p* < 0.01). Furthermore, anxiety exerted a significant negative influence on these same dimensions: Social Psychological Function (*β* = −6.111, *p* < 0.01), Physical and Psychological Health (*β* = −4.352, *p* < 0.01), Living Environment (*β* = −2.158, *p* < 0.01), and QoL Satisfaction (*β* = −3.292, *p* < 0.01). As shown in Table 4, the mediation effects of anxiety across the four QoL dimensions were all statistically significant: Social Psychological Function: 14.429% (95% CI: 0.020, 0.038), Physical and Psychological Health: 59.128% (95% CI: 0.021, 0.045), Living Environment: 6.102% (95% CI: 0.015, 0.029), QoL Satisfaction: 13.271% (95% CI: 0.024, 0.047). In every instance, the confidence intervals excluded zero, which suggests a significant mediating role of anxiety. The moderating effects of gender are outlined in the Supplementary Tables 7–10: gender exerted a negative moderating influence on the relationship between anxiety and Social Psychological Function (*β* = −6.111, *p* < 0.01), gender negatively moderated the link between anxiety and Physical and Psychological Health (*β* = −0.503, *p* < 0.05), gender positively moderated the association between physical activity and Physical and Psychological Health (*β* = 0.729, *p* < 0.01), and gender positively moderated the connection between anxiety and Living Environment (*β* = 0.536, *p* < 0.01). Nevertheless, gender did not play a significant moderating role in the relationship between anxiety and QoL Satisfaction. The moderated mediation model for multidimensional QoL is depicted in Figure 6.

## 4 Discussion

This study investigated the predictive effect of physical activity on QoL among adolescents and examined the potential mediating role of anxiety. The results indicated that engaging in physical activity significantly reduces anxiety, thereby positively influencing QoL. Increased physical activity levels showed a correlation with reduced anxiety and better QoL, supporting Hypotheses 1 and 2. In addition, the study demonstrated that gender significantly influenced the relationships between physical activity and QoL, as well as between anxiety and QoL, lending credence to Hypotheses 3 and 5. However, no significant moderating effect of gender was observed between physical activity and anxiety; hence, Hypothesis 4 was not supported. For both male and female adolescents, increased physical activity was associated with improved QoL, with a more pronounced effect observed among females. In contrast, anxiety had a stronger negative impact on QoL in males. A multidimensional analysis of QoL was also conducted. The results showed that gender significantly moderated the relationships between anxiety and social-psychological functioning, anxiety and physical-psychological health, anxiety and living environment, and between physical activity and physical-psychological health. However, no significant moderating effect of gender was observed on the association between anxiety and QoL Satisfaction.

### 4.1 Relationship between physical activity and quality of life

This study confirms the positive relationship between adolescent physical activity and QoL, consistent with existing evidence demonstrating that regular exercise enhances life satisfaction (7, 55). Importantly, these results substantiate a central tenet of Social Cognitive Theory—the mediating role of self-efficacy (56). Specifically, participation in physical activities promotes subjective well-being by strengthening adolescents’ confidence in their physical control and emotional regulation, such as through enhanced motor skills and decreased frustration-related anxiety (57). Furthermore, this finding represents a significant extension of Self-Determination Theory (58). When physical activities satisfy adolescents’ need for autonomy, sustained engagement is more likely to foster a reinforcing cycle of self-efficacy (59, 60), which can only be fully translated into improved QoL when anxiety is effectively reduced. This may explain the limited effectiveness observed in interventions that solely emphasize the general benefits of physical activity (61). Consequently, future health promotion strategies have the potential to achieve meaningful theoretical and practical advancements by prioritizing consistent participation, embedding progressive efficacy-building objectives, and integrating targeted anxiety management techniques within sport-based interventions (62), thereby maximizing QoL improvement.

### 4.2 Mediating role of anxiety

Modern adolescents commonly face psychological difficulties, including anxiety (63), which not only disrupt their developmental pathways (64) but also markedly degrade QoL by adversely affecting both mental and physical well-being (65) and impairing social functioning (66). Across the globe, youth are identified as a high-risk group in need of customized mental health interventions (16). Our findings indicate that engaging in physical activity can alleviate anxiety and improve QoL through a dual-mechanism process. These results further substantiate the positive association between physical activity and mental health, indicating that increased participation in exercise correlates with enhanced psychological well-being and reduced anxiety levels (62). Establishing regular physical activity patterns during adolescence not only elevates subjective well-being but also promotes mental health by mitigating symptoms of anxiety (67), stress, and depression, thereby leading to improved emotional and behavioral regulation (68, 69). At the neurobiological level, exercise supports the regulation of the hypothalamic–pituitary–adrenal (HPA) axis and stimulates neural plasticity, thus targeting the neurological origins of anxiety (31). From a psychosocial angle, involvement in team-based sports such as basketball and soccer fosters social inclusion, allowing young people to restore their self-confidence through cooperative engagement (32). The interplay between these biological and psychological processes converts anxiety reduction into observable enhancements in QoL.

It is important to recognize that the success of these mechanisms is largely shaped by local cultural values and socio-economic environments. In Shandong’s Confucian-based educational framework, rigorous academic expectations limit the time adolescents typically spend on physical activities. Nonetheless, traditional group exercises like martial arts and communal workouts can draw upon culturally significant concepts—such as collective identity—to strengthen youths’ sense of personal capability (70). Economic inequality further widens the gap due to unequal distribution of resources; families with limited income may struggle with anxiety management owing to scarce access to public recreational facilities and insufficient awareness of the benefits of physical activity (71).

Hence, embedding physical activity as a central approach for anxiety prevention should start at the neurophysiological level by introducing high-intensity interval training (HIIT) in middle schools; research indicates that just one 20-min session can improve HPA axis function (72). Secondly, from a cultural integration perspective, brief, school-based physical activities—such as five-minute seated movement breaks—should be embedded into the daily schedule, while promoting collectivist principles through team-oriented reward systems (32, 73). Finally, to promote equity across different socio-economic levels, low-cost and community-centered sports initiatives must be expanded for underserved groups, including equipment-sharing programs, and formal channels for school team involvement should be created to remove participation obstacles.

### 4.3 Moderating role of gender

Theoretical frameworks suggest that gender differences in physical activity are shaped by biological, psychological, cognitive, and socio-developmental factors (74). Gender significantly influences both motivation and outcomes in physical activity, which may relate to psychological needs, participation motives, and satisfaction levels (75, 76). Due to inherent physiological differences, males typically exhibit greater spontaneity, engagement, and competence in physical activity (76, 77). However, with increasing participation, females report greater emotional, social, and self-perceptual satisfaction from physical activity (78). Long-term health benefits for females include higher activity levels, lower BMI, and reduced obesity risk (79).

Meta-analyses show that females experience greater QoL improvements from physical activity, especially in the domains of physical functioning and social roles (80), indicating that its indirect effects are more pronounced among females (81, 82). Simultaneously, females demonstrate a greater capacity to utilize social interactions in team sports to alleviate anxiety. Evidence also suggests that females experience lower levels of anxiety than males (83). Males exhibit higher levels of anxiety and depression, both of which strongly affect their QoL (84). Anxiety-related declines in QoL are common among young males (85), and studies among college students have found that males have lower psychological and social QoL when anxiety is clinically significant. Research in China has also noted that males are more susceptible to psychological stressors, leading to reduced QoL (86). These findings align with the current study: physical activity improves QoL more significantly for females, whereas anxiety more severely compromises QoL for males.

This natural physiological tendency is further influenced by sociocultural norms. In particular, dominant societal expectations often push men to display “masculinity” through competitive achievement, which may restrict emotional openness and cause unaddressed anxiety to manifest as physical symptoms, thus impairing QoL (87). On the other hand, the sense of connection that women gain from collaborative activities can act as a buffer against stressors tied to academic pressure and appearance-related issues (88); however, unequal access to resources hampers the full realization of these benefits (89). These outcomes correspond with the findings of the present research, which suggest that physical activity has a more favorable effect on women’s QoL, while men are more likely to suffer from anxiety-related negative impacts. Based on these gender-specific mechanisms (90), healthcare practices should consider targeted interventions—embedding emotional regulation techniques into male-oriented competitive sports (91) and providing affordable, group-focused programs designed for women (92).

### 4.4 Multidimensional analysis of quality of life

Earlier research has demonstrated that engaging in physical activity may lower anxiety levels, leading to enhancements in various dimensions of QoL—such as physical-psychological health, social relationships, and environmental adaptation—among adolescents (93, 94). Anxiety serves as a significant mediator, confirming its central role in the physical activity—QoL relationship (95). Our results align with this notion, indicating that physical activity enhances four dimensions of QoL—social-psychological functioning, physical-psychological health, living environment, and satisfaction with QoL—by mediating the effect through anxiety. Notably, gender did not play a significant moderating role in the association between anxiety and QoL satisfaction. In contrast, gender exhibited a negative moderating effect on the relationship between anxiety and physical-psychological health. Among the various moderated mediation effects, only the dimension of physical-psychological health exhibited a significant moderating effect of gender. This may be attributed to the interaction between biological responses and social role expectations. Some studies suggest that QoL Satisfaction is influenced more by individual subjectivity and less by gender (96). Anxiety more strongly affects physical-psychological health, social functioning, and environmental dimensions (83), with its impact on female QoL being especially pronounced in health-related outcomes (97). Other studies support our finding that gender differences primarily appear in the effects of physical activity on physical and psychological health, while effects on social and environmental dimensions are not significantly moderated by gender (98, 99).

However, this emphasizes the importance of future studies focusing on uncovering the mechanisms involved in this particular dimension. As a result, upcoming research should incorporate more detailed variables, such as specific neurobiological markers (100), objective indicators of physiological stress responses (101), and in-depth analyses of sociocultural norms (102). Moreover, utilizing advanced research methodologies—including longitudinal study designs (103), real-time physiological measurements (104)—can greatly improve the rigor of such investigations. These methodological improvements will facilitate a more accurate understanding of how biological and sociocultural elements interact dynamically through gender-specific pathways in the realm of physical-psychological health. This area of research is anticipated to shed light on the underlying causes of existing disparities and guide the creation of focused, evidence-driven intervention strategies. In the long run, thorough investigation in this field may lead to the identification of more gender-responsive methods for promoting equitable health outcomes.

### 4.5 Limitations

This research examined the mediating effect of anxiety on the relationship between physical activity and QoL among adolescents and assessed the moderating influence of gender. A comprehensive multidimensional analysis of QoL was also conducted. The large and diverse sample provides broad representativeness, offering a realistic reflection of adolescents’ experiences. The findings offer new insights into adolescent health promotion mechanisms and provide empirical support for differentiated intervention strategies.

Although this study has notable strengths, several limitations should be considered in future research. First, the cross-sectional design identifies associations but does not support causal conclusions. Therefore, the proposed pathway—"physical activity → reduced anxiety → improved quality of life”—remains speculative. While statistical methods were used to examine mediation and moderation, unmeasured variables or alternative models may also explain the results. The actual mechanisms may be more complex than identified. Second, anxiety was measured only with the SCL-90 anxiety subscale, which captures emotional distress but misses key physical and behavioral symptoms—such as somatic discomfort and avoidance—that are central to anxiety disorders. This limits understanding of how anxiety interacts with physical activity and QoL. Physical activity was assessed through self-report (PAQ-A), which is prone to recall errors and social desirability bias, possibly leading to overestimation. In addition, data collected at a single time point cannot reflect dynamic changes or long-term trends. Third, although the sample was large and representative within Shandong Province, findings may not generalize to other regions or countries due to differences in culture, economy, and education. Studies in diverse contexts are needed to improve external validity. Also, since participants were aged 12–18, results may not apply to younger children or young adults. Lastly, while gender was examined as a moderator, other factors such as being an only child, parental education, and family socioeconomic status were included only as controls. Their potential moderating effects were not analyzed. Future studies should explore these variables in depth to better understand contextual influences on the studied relationships.

## 5 Conclusion

In summary, the following conclusions can be drawn: Adolescent physical activity significantly enhances QoL. Active participation in physical exercise is an effective means to promote overall adolescent well-being. Engaging in physical activity enhances QoL both directly and indirectly through the reduction of anxiety. Increased physical activity correlates with decreased anxiety, which subsequently leads to an improvement in QoL. The influence of physical activity and anxiety on QoL varies across genders, as gender acts as a moderating factor in this relationship. Female adolescents experience more substantial improvements in happiness and QoL through physical activity, while male adolescents are more vulnerable to anxiety-related declines in QoL. The impact of physical activity on different QoL dimensions is mediated by anxiety and moderated by gender, indicating the presence of both universal and gender-specific mechanisms. These findings provide a theoretical foundation for using physical activity as an intervention to improve adolescent mental health and QoL and highlight the importance of gender-sensitive strategies in policy and practice.

## Data Availability

The datasets presented in this study can be found in online repositories. The names of the repository/repositories and accession number(s) can be found at: https://www.ncmi.cn/phda/dataDetails.do?id=CSTR:17970.11.A0031.202107.209.V1.0.
