# Supplementary material for: The impact of adolescent physical activity on quality of life: a moderated mediation model
Source: Front Public Health. 2025 Jul 18;13:1625066. doi: 10.3389/fpubh.2025.1625066 (PMC12313584; doi:10.3389/fpubh.2025.1625066)
Supplement: Supplementary file 1 [file Table_1.DOCX]

TABLE 1 Analysis of variance of the main variables.

| **Control Variables** | **Options** | **Quality of life** | **p-value** | **Physical Activity** | **p-value** | **Anxiety** | **p-value** |
| --- | --- | --- | --- | --- | --- | --- | --- |
| Gender | Male | 137.10±20.79 | 0.000** | 4.27±1.48 | 0.000** | 1.51±0.59 | 0.000** |
|  | Female | 135.13±19.27 |  | 3.71±1.16 |  | 1.60±0.61 |  |
| Address | urban | 138.25±21.01 | 0.000** | 4.02±1.42 | 0.001** | 1.53±0.62 | 0.000** |
|  | non-urban | 134.14±18.93 |  | 3.93±1.29 |  | 1.58±0.58 |  |
| Only child | Yes | 136.46±21.49 | 0.223 | 4.13±1.47 | 0.000** | 1.53±0.60 | 0.003** |
|  | No | 135.91±19.39 |  | 3.91±1.29 |  | 1.57±0.60 |  |
| Father's education level | Below high school | 134.46±19.78 | 0.000** | 3.90±1.31 | 0.000** | 1.59±0.62 | 0.000** |
|  | From high school to undergraduate | 136.80±19.39 |  | 4.17±1.40 |  | 1.55±0.59 |  |
|  | Bachelor's degree or above | 140.80±20.79 |  | 4.01±1.42 |  | 1.47±0.55 |  |
| Mother's education level | Below high school | 134.74±19.66 | 0.000** | 3.91±1.30 | 0.000** | 1.58±0.61 | 0.000** |
|  | From high school to undergraduate | 137.28±20.10 |  | 4.15±1.41 |  | 1.53±0.59 |  |
|  | Bachelor's degree or above | 141.07±20.84 |  | 4.07±1.48 |  | 1.46±0.55 |  |
| Family economic condition | Very difficult | 118.46±25.01 | 0.000** | 4.31±1.65 | 0.000** | 1.94±0.95 | 0.000** |
|  | Rather difficult | 130.32±19.66 |  | 3.77±1.21 |  | 1.68±0.65 |  |
|  | Medium | 137.39±18.96 |  | 3.94±1.30 |  | 1.52±0.56 |  |
|  | Relatively wealthy | 143.18±21.42 |  | 4.46±1.65 |  | 1.53±0.64 |  |
|  | Very rich | 128.11±25.43 |  | 4.92±1.87 |  | 1.74±0.73 |  |

N=9,504,* p<0.05 ** p<0.01.

TABLE 2 Analysis of variance effect results (N=9,504)

| **Control Variables** | **Partial η2** | | |
| --- | --- | --- | --- |
|  | **Quality of life** | **Anxiety** | **Physical Activity** |
| Gender | 0.002 | 0.003 | 0.008 |
| Address | 0.010 | 0.015 | 0.007 |
| Only child | 0.000 | 0.001 | 0.005 |
| Father's education level | 0.014 | 0.005 | 0.006 |
| Mother's education level | 0.013 | 0.005 | 0.005 |
| Family economic condition | 0.049 | 0.023 | 0.021 |

TABLE 3 Results of hierarchical regression analysis (N=9,504)

|  | **Social Psychological Function** | | | **Anxiety** | | | **Social Psychological Function** | | |
| --- | --- | --- | --- | --- | --- | --- | --- | --- | --- |
|  | **B** | **SE** | **t** | **B** | **SE** | **t** | **B** | **SE** | **t** |
| Constant | 41.792** | 0.855 | 48.875 | 1.986** | 0.050 | 39.525 | 53.927** | 0.861 | 62.615 |
| Only child | 1.929** | 0.239 | 8.069 | 0.005 | 0.014 | 0.341 | 1.958** | 0.223 | 8.776 |
| Father's education level | 0.740** | 0.172 | 4.306 | -0.027** | 0.010 | -2.710 | 0.572** | 0.160 | 3.569 |
| Mother's education level | 0.416* | 0.185 | 2.250 | -0.021 | 0.011 | -1.944 | 0.287 | 0.172 | 1.663 |
| Family economic condition | 2.102** | 0.175 | 12.006 | -0.078** | 0.010 | -7.606 | 1.624** | 0.164 | 9.907 |
| Address | -0.439* | 0.224 | -1.961 | 0.010 | 0.013 | 0.765 | -0.377 | 0.209 | -1.806 |
| Physical activity | 1.607** | 0.077 | 20.803 | -0.038** | 0.005 | -8.360 | 1.375** | 0.072 | 19.002 |
| Anxiety |  |  |  |  |  |  | -6.111** | 0.163 | -37.488 |
| R 2 | 0.074 | | | 0.021 | | | 0.194 | | |
| F | F (6,9497)=126.999,p=0.000 | | | F (6,9497)=33.483,p=0.000 | | | F (7,9496)=325.722,p=0.000 | | |

* p<0.05 ** p<0.01.

TABLE 4 Results of hierarchical regression analysis (N=9,504)

|  | **Physical and Psychological Health** | | | **Anxiety** | | | **Physical and Psychological Health** | | |
| --- | --- | --- | --- | --- | --- | --- | --- | --- | --- |
|  | **B** | **SE** | **t** | **B** | **SE** | **t** | **B** | **SE** | **t** |
| Constant | 33.974** | 0.536 | 63.352 | 1.986** | 0.050 | 39.525 | 42.617** | 0.528 | 80.646 |
| Only child | -0.238 | 0.150 | -1.587 | 0.005 | 0.014 | 0.341 | -0.217 | 0.137 | -1.585 |
| Father's education level | -0.044 | 0.108 | -0.413 | -0.027** | 0.010 | -2.710 | -0.164 | 0.098 | -1.662 |
| Mother's education level | 0.209 | 0.116 | 1.801 | -0.021 | 0.011 | -1.944 | 0.117 | 0.106 | 1.104 |
| Family economic condition | -0.177 | 0.110 | -1.616 | -0.078** | 0.010 | -7.606 | -0.518** | 0.101 | -5.150 |
| Address | -0.734** | 0.140 | -5.233 | 0.010 | 0.013 | 0.765 | -0.690** | 0.128 | -5.389 |
| Physical activity | 0.279** | 0.048 | 5.765 | -0.038** | 0.005 | -8.360 | 0.114* | 0.044 | 2.571 |
| Anxiety |  |  |  |  |  |  | -4.352** | 0.100 | -43.514 |
| R 2 | 0.009 | | | 0.021 | | | 0.174 | | |
| F | F (6,9497)=14.417,p=0.000 | | | F (6,9497)=33.483,p=0.000 | | | F (7,9496)=285.321,p=0.000 | | |

* p<0.05 ** p<0.01.

TABLE 5 Results of hierarchical regression analysis (N=9,504)

|  | **Living Environment** | | | **Anxiety** | | | **Living Environment** | | |
| --- | --- | --- | --- | --- | --- | --- | --- | --- | --- |
|  | **B** | **SE** | **t** | **B** | **SE** | **t** | **B** | **SE** | **t** |
| Constant | 13.658** | 0.350 | 39.045 | 1.986** | 0.050 | 39.525 | 17.944** | 0.359 | 49.997 |
| Only child | 0.187 | 0.098 | 1.910 | 0.005 | 0.014 | 0.341 | 0.197* | 0.093 | 2.120 |
| Father's education level | 0.417** | 0.070 | 5.938 | -0.027** | 0.010 | -2.710 | 0.358** | 0.067 | 5.359 |
| Mother's education level | 0.148 | 0.076 | 1.954 | -0.021 | 0.011 | -1.944 | 0.102 | 0.072 | 1.421 |
| Family economic condition | 1.109** | 0.072 | 15.488 | -0.078** | 0.010 | -7.606 | 0.940** | 0.068 | 13.768 |
| Address | -1.085** | 0.091 | -11.855 | 0.010 | 0.013 | 0.765 | -1.063** | 0.087 | -12.219 |
| Physical activity | 1.342** | 0.032 | 42.473 | -0.038** | 0.005 | -8.360 | 1.260** | 0.030 | 41.792 |
| Anxiety |  |  |  |  |  |  | -2.158** | 0.068 | -31.774 |
| R 2 | 0.219 | | | 0.021 | | | 0.294 | | |
| F | F (6,9497)=444.182,p=0.000 | | | F (6,9497)=33.483,p=0.000 | | | F (7,9496)=565.391,p=0.000 | | |

* p<0.05 ** p<0.01.

TABLE 6 Results of hierarchical regression analysis (N=9,504)

|  | **Quality of Life Satisfaction** | | | **Anxiety** | | | **Quality of Life Satisfaction** | | |
| --- | --- | --- | --- | --- | --- | --- | --- | --- | --- |
|  | **B** | **SE** | **t** | **B** | **SE** | **t** | **B** | **SE** | **t** |
| Constant | 15.976** | 0.358 | 44.648 | 1.986** | 0.050 | 39.525 | 22.513** | 0.342 | 65.749 |
| Only child | 0.195 | 0.100 | 1.946 | 0.005 | 0.014 | 0.341 | 0.210* | 0.089 | 2.372 |
| Father's education level | 0.238** | 0.072 | 3.316 | -0.027** | 0.010 | -2.710 | 0.148* | 0.064 | 2.326 |
| Mother's education level | 0.109 | 0.077 | 1.408 | -0.021 | 0.011 | -1.944 | 0.039 | 0.069 | 0.575 |
| Family economic condition | 0.911** | 0.073 | 12.440 | -0.078** | 0.010 | -7.606 | 0.654** | 0.065 | 10.032 |
| Address | -0.272** | 0.094 | -2.905 | 0.010 | 0.013 | 0.765 | -0.239** | 0.083 | -2.877 |
| Physical activity | 0.941** | 0.032 | 29.120 | -0.038** | 0.005 | -8.360 | 0.816** | 0.029 | 28.375 |
| Anxiety |  |  |  |  |  |  | -3.292** | 0.065 | -50.800 |
| R 2 | 0.110 | | | 0.021 | | | 0.300 | | |
| F | F (6,9497)=195.958,p=0.000 | | | F (6,9497)=33.483,p=0.000 | | | F (7,9496)=582.245,p=0.000 | | |

* p<0.05 ** p<0.01.

TABLE 7 Test on the moderating effect of gender(N=9,504)

|  | **Social Psychological Function** | | | | | | **Anxiety** | | | | | | |
| --- | --- | --- | --- | --- | --- | --- | --- | --- | --- | --- | --- | --- | --- |
|  | **β** | **SE** | **t** | | **p** | | **β** | | **SE** | | **t** | | **p** |
| Constant | 54.041 | 1.490 | 36.260 | | 0.000** | | 1.925 | | 0.074 | | 25.860 | | 0.000** |
| Physical activity | 1.510 | 0.222 | 6.796 | | 0.000** | | -0.042 | | 0.014 | | -3.010 | | 0.003** |
| Anxiety | -7.642 | 0.527 | -14.491 | | 0.000** | |  | |  | |  | |  |
| Gender | 0.172 | 0.831 | 0.207 | | 0.836 | | 0.043 | | 0.039 | | 1.102 | | 0.270 |
| Only child | 1.672 | 0.225 | 7.419 | | 0.000** | | -0.007 | | 0.014 | | -0.518 | | 0.605 |
| Father's education level | 0.606 | 0.160 | 3.788 | | 0.000** | | -0.026 | | 0.010 | | -2.599 | | 0.009** |
| Mother's education level | 0.248 | 0.172 | 1.441 | | 0.150 | | -0.023 | | 0.011 | | -2.101 | | 0.036* |
| Family economic condition | 1.569 | 0.163 | 9.595 | | 0.000** | | -0.080 | | 0.010 | | -7.761 | | 0.000** |
| Address | -0.386 | 0.208 | -1.855 | | 0.064 | | 0.010 | | 0.013 | | 0.726 | | 0.468 |
| Physical activity * Gender | -0.022 | 0.150 | -0.145 | | 0.885 | | 0.006 | | 0.009 | | 0.687 | | 0.492 |
| Anxiety*Gender | 0.947 | 0.325 | 2.914 | 0.004** | |  | |  | |  | |  | |
| R 2 | 0.199 | | | | | 0.024 | | | | | | | |
| F | F (10,9493)=236.474,p=0.000 | | | | | F (8,9495)=28.918,p=0.000 | | | | | | | |

* p<0.05 ** p<0.01.

TABLE 8 Test on the moderating effect of gender(N=9,504)

|  | **Physical and Psychological Health** | | | | **Anxiety** | | | |
| --- | --- | --- | --- | --- | --- | --- | --- | --- |
|  | **β** | **SE** | **t** | **p** | **β** | **SE** | **t** | **p** |
| Constant | 45.393 | 0.914 | 49.649 | 0.000** | 1.925 | 0.074 | 25.860 | 0.000** |
| Physical activity | -0.901 | 0.136 | -6.611 | 0.000** | -0.042 | 0.014 | -3.010 | 0.003** |
| Anxiety | -3.583 | 0.324 | -11.076 | 0.000** |  |  |  |  |
| Gender | -2.056 | 0.510 | -4.033 | 0.000** | 0.043 | 0.039 | 1.102 | 0.270 |
| Only child | -0.192 | 0.138 | -1.386 | 0.166 | -0.007 | 0.014 | -0.518 | 0.605 |
| Father's education level | -0.171 | 0.098 | -1.741 | 0.082 | -0.026 | 0.010 | -2.599 | 0.009** |
| Mother's education level | 0.122 | 0.105 | 1.155 | 0.248 | -0.023 | 0.011 | -2.101 | 0.036* |
| Family economic condition | -0.510 | 0.100 | -5.082 | 0.000** | -0.080 | 0.010 | -7.761 | 0.000** |
| Address | -0.675 | 0.128 | -5.291 | 0.000** | 0.010 | 0.013 | 0.726 | 0.468 |
| Physical activity * Gender | 0.729 | 0.092 | 7.944 | 0.000** | 0.006 | 0.009 | 0.687 | 0.492 |
| Anxiety*Gender | -0.503 | 0.199 | -2.522 | 0.012* |  |  |  |  |
| R 2 | 0.180 | | | | 0.024 | | | |
| F | F (10,9493)=208.523,p=0.000 | | | | F (8,9495)=28.918,p=0.000 | | | |

* p<0.05 ** p<0.01.

TABLE 9 Test on the moderating effect of gender(N=9,504)

|  | **Living Environment** | | | | **Anxiety** | | | |
| --- | --- | --- | --- | --- | --- | --- | --- | --- |
|  | **β** | **SE** | **t** | **p** | **β** | **SE** | **t** | **p** |
| Constant | 19.890 | 0.623 | 31.949 | 0.000** | 1.925 | 0.074 | 25.860 | 0.000** |
| Physical activity | 1.155 | 0.093 | 12.441 | 0.000** | -0.042 | 0.014 | -3.010 | 0.003** |
| Anxiety | -2.976 | 0.220 | -13.511 | 0.000** |  |  |  |  |
| Gender | -1.305 | 0.347 | -3.761 | 0.000** | 0.043 | 0.039 | 1.102 | 0.270 |
| Only child | 0.239 | 0.094 | 2.542 | 0.011* | -0.007 | 0.014 | -0.518 | 0.605 |
| Father's education level | 0.359 | 0.067 | 5.371 | 0.000** | -0.026 | 0.010 | -2.599 | 0.009** |
| Mother's education level | 0.110 | 0.072 | 1.532 | 0.126 | -0.023 | 0.011 | -2.101 | 0.036* |
| Family economic condition | 0.938 | 0.068 | 13.735 | 0.000** | -0.080 | 0.010 | -7.761 | 0.000** |
| Address | -1.055 | 0.087 | -12.141 | 0.000** | 0.010 | 0.013 | 0.726 | 0.468 |
| Physical activity * Gender | 0.061 | 0.062 | 0.983 | 0.326 | 0.006 | 0.009 | 0.687 | 0.492 |
| Anxiety*Gender | 0.536 | 0.136 | 3.952 | 0.000** |  |  |  |  |
| R 2 | 0.296 | | | | 0.024 | | | |
| F | F (10,9493)=398.983,p=0.000 | | | | F (8,9495)=28.918,p=0.000 | | | |

* p<0.05 ** p<0.01.

TABLE 10 Test on the moderating effect of gender(N=9,504)

|  | **Quality of Life Satisfaction** | | | | | **Anxiety** | | | |
| --- | --- | --- | --- | --- | --- | --- | --- | --- | --- |
|  | **β** | **SE** | **t** | **p** | | **β** | **SE** | **t** | **p** |
| Constant | 23.664 | 0.594 | 39.805 | 0.000** | | 1.925 | 0.074 | 25.860 | 0.000** |
| Physical activity | 0.674 | 0.089 | 7.604 | 0.000** | | -0.042 | 0.014 | -3.010 | 0.003** |
| Anxiety | -3.594 | 0.210 | -17.085 | 0.000** | |  |  |  |  |
| Gender | -0.790 | 0.331 | -2.384 | 0.017* | | 0.043 | 0.039 | 1.102 | 0.270 |
| Only child | 0.233 | 0.090 | 2.590 | 0.010** | | -0.007 | 0.014 | -0.518 | 0.605 |
| Father's education level | 0.148 | 0.064 | 2.318 | 0.020* | | -0.026 | 0.010 | -2.599 | 0.009** |
| Mother's education level | 0.044 | 0.069 | 0.636 | 0.525 | | -0.023 | 0.011 | -2.101 | 0.036* |
| Family economic condition | 0.654 | 0.065 | 10.022 | 0.000** | | -0.080 | 0.010 | -7.761 | 0.000** |
| Address | -0.234 | 0.083 | -2.818 | 0.005** | | 0.010 | 0.013 | 0.726 | 0.468 |
| Physical activity * Gender | 0.095 | 0.060 | 1.600 | 0.110 | | 0.006 | 0.009 | 0.687 | 0.492 |
| Anxiety*Gender | 0.198 | 0.130 | 1.529 | 0.126 |  | |  |  |  |
| R 2 | 0.301 | | | | 0.024 | | | | |
| F | F (10,9493)=408.342,p=0.000 | | | | F (8,9495)=28.918,p=0.000 | | | | |

* p<0.05 ** p<0.01.
